# Work fatigue during COVID-19 lockdown teleworking: the role of psychosocial, environmental, and social working conditions

**DOI:** 10.3389/fpsyg.2023.1155118

**Published:** 2023-05-16

**Authors:** Clara Weber, Sarah E. Golding, Joanna Yarker, Kevin Teoh, Rachel Lewis, Eleanor Ratcliffe, Fehmidah Munir, Theresa Wheele, Lukas Windlinger

**Affiliations:** ^1^Institute of Facility Management, School of Life Sciences and Facility Management, Zurich University of Applied Sciences, Zurich, Switzerland; ^2^School of Psychology, Faculty of Health and Medical Sciences, University of Surrey, Guildford, United Kingdom; ^3^Department of Organisational Psychology, Birkbeck University of London, London, United Kingdom; ^4^School of Sport, Exercise and Health Sciences, Loughborough University, Loughborough, United Kingdom

**Keywords:** COVID-19, teleworking, home office, office design, privacy, psychosocial working conditions, lockdown, burnout

## Abstract

**Background:**

During national lockdowns in response to the COVID-19 pandemic, previously office-based workers who transitioned to home-based teleworking faced additional demands (e.g., childcare, inadequate homeworking spaces) likely resulting in poor work privacy fit. Previous office research suggests poor work privacy fit is associated with lower wellbeing and higher work fatigue. Emerging evidence suggests a relationship between childcare duties during pandemic teleworking and work fatigue. In addition to psychosocial working conditions (job demand, job control, and job change management), which are acknowledged predictors of work fatigue, this poses a significant threat to occupational health during pandemic teleworking. However, the relative effects of aspects of the psychosocial environment (job demands and resources), the home office environment (including privacy fit), and the social environment (childcare) on work fatigue as well as their interactions are under-explored.

**Objective:**

This study examined the relationships between the psychosocial, environmental, and social working conditions of teleworking during the first COVID-19 lockdown and work fatigue. Specifically, the study examined teleworkers’ physical work environment (e.g., if and how home office space is shared, crowding, and noise perceptions) as predictors of privacy fit and the relationship between privacy fit, childcare, psychosocial working conditions (job demand, job control, and job change management), and work fatigue. Work privacy fit was hypothesized to mediate the relationship between childcare and work fatigue.

**Methods:**

An online cross-sectional survey was conducted with teleworkers (*n* = 300) during the first COVID-19 lockdown in April and May 2020; most participants were in Germany, Switzerland, and the United Kingdom.

**Results:**

Path analysis was used to examine the hypothesized relationships. Privacy fit was lower for those reporting greater levels of noise in home-working spaces and those feeling crowded at home. Work fatigue was lower amongst those with greater privacy fit and higher amongst those with high levels of job demand. An indirect relationship was observed between childcare and work fatigue with privacy fit mediating this relationship.

**Conclusion:**

The influence of privacy fit has so far been largely neglected in research on teleworking, especially during the pandemic. However, its contribution to workers’ wellbeing should be acknowledged in occupational health strategies.

## Introduction

The COVID-19 pandemic and its social distancing measures have resulted in dramatic changes to working life for many sectors and roles. These include job insecurity, job loss, job changes, and/or reduced control over job roles and responsibilities as organisations pivot to different business models (Restubog et al., 2020). Due to national lockdowns and other pandemic restrictions, many individuals who typically worked in offices or other communal settings had to rapidly switch to teleworking from home. Despite the general flexibilities offered by telework, the sudden shift to extreme teleworking in newly-created home offices likely led to new and intense strains in job roles, in the physical working environments and in the social context at home. These sudden changes required new or different levels of resources and pose a likely risk to occupational health, such as work fatigue.

Work fatigue (also referred to as exhaustion, *cf.* Frone and Tidwell, 2015) is central to job burnout theories (e.g., Job Demands-Resources (JDR) model, conservation of resources (COR) theory; Hobfoll, 1989; Maslach et al., 1997; Demerouti et al., 2001; Shirom, 2003). Job burnout theories describe an energy depletion-protection/renewal process through job demands and job resources. Demands require sustained effort that depletes energy, resulting in emotional, cognitive/mental, and physical work fatigue, whereas resources can protect or renew energy. Interest in the drivers and consequences of work fatigue has risen in recent decades given its links to employee health, motivation, and performance (Frone and Tidwell, 2015). Work fatigue and burnout research during the pandemic has predominantly focused on specific occupations such as frontline healthcare workers (Azoulay et al., 2020; Barello et al., 2020; Matsuo et al., 2020) and teachers (Panisoara et al., 2020; Sokal et al., 2020; Daumiller et al., 2021) as the pandemic has placed considerable psychological strain on members of these professions. Increasing attention is, however, being given to teleworker fatigue and burnout (Abdel Hadi et al., 2021; Barriga Medina et al., 2021). Although relationships between job demands, job resources, and work fatigue in a non-pandemic context are established, some emerging evidence suggests that prior knowledge cannot be readily transferred, since pandemic working poses new issues and intensifies existing issues (Wang et al., 2020). Furthermore, the impact of relevant job resources, such as job control and job change management, appears underexplored in pandemic teleworking research.

Teleworking research during the pandemic has mostly examined the blurred lines between job and private domains, specifically childcare, and health consequences such as work fatigue (Wang et al., 2020; Abdel Hadi et al., 2021; Barriga Medina et al., 2021; Da et al., 2022). Comparatively little research has considered the impact of the physical teleworking environment on work fatigue. However, as pandemic research indicates, homeworking environments varied drastically across countries and sectors (European Commission, 2020), resulting in privacy-related issues and risking teleworkers’ health. Lack of privacy is a huge health and performance concern in office research (*cf.* Weber et al., 2021). We focus in this study on work privacy fit, which is rooted in Person-Environment fit theory and describes the congruence between the desired and the actual level of work privacy. Work privacy is defined as a socio-environmental control process of information and social stimuli in the work environment. Workers attempt to achieve the best possible fit between their actual and desired levels of input/stimuli from their colleagues and output they make to their colleagues. As outlined in the privacy fit theory, work- and health-related outcomes can be maximised if environmental characteristics can be organised in a way that supports individual privacy needs (Weber, 2019; Weber et al., 2021). However, work privacy fit and its predictors during pandemic teleworking has been almost neglected.

As pre-pandemic and pandemic studies have indicated separate relationships between job demands and job resources, the home office environment, privacy-related issues, childcare, and work fatigue, our study examined relationships together between these factors during the first COVID-19 lockdown in 2020. Our study makes three key contributions to the existing literature:

1. Examines the relationship between the physical environment (home office characteristics) and privacy fit.
2. Examines the relative effect of psychosocial (job demands and resources), environmental (privacy fit), and social (childcare) working conditions on work fatigue.
3. Examines the interaction between environmental (work privacy fit) and social (childcare) working conditions and work fatigue.

**Figure 1:**
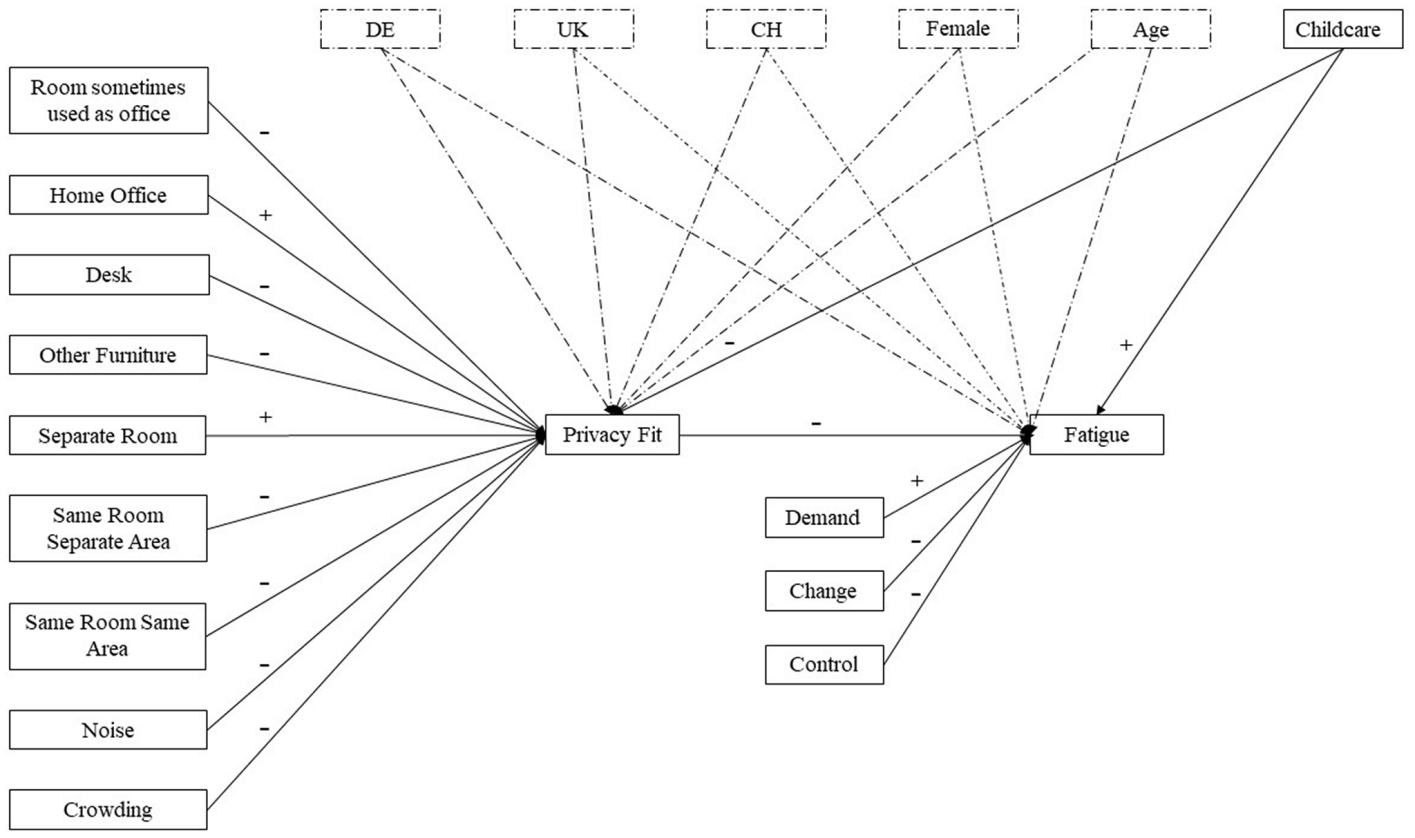
Conceptual model. Control variables are indicated by dashed lines. CH, Switzerland; DE, Germany; UK, United Kingdom.

The conceptual model of our study is presented in Figure 1.

### Overarching theoretical approach: socio-ecological framework

We examine psychosocial, environmental, and social teleworking conditions during lockdown and their relationship to work fatigue through the theoretical lens of the socio-ecological framework on work context (Stokols, 1996; Sallis and Owen, 2015; Munir et al., 2021). This theory-based framework suggests that health at work is influenced by factors across four nested levels: (1) individual determinants; (2) social environments; (3) built environments; and (4) structural environments. In this study we examined how factors across the levels of this framework influence work fatigue. We also examined interactions between these factors. Starting at the fourth, outer level, the framework proposes that health at work is influenced by structural factors, such as job design and teleworking policies. We examined the role of psychosocial working conditions during lockdown telework (job control, job demand, job change) as structural factors. At the third level, health is influenced by the built environment and its (in) adequacy to meet individuals’ work needs. We examined aspects of the home office (setup and privacy fit) as built environment factors. At the second level, health is influenced by the social network that operates within an environment. We examined social family presence when teleworking, specifically childcare duties, as a social environment factor. Finally, as health is also influenced by individuals’ demographic characteristics, we included age and gender as individual-level control variables. We also included country of residence during lockdown as a structural-level control variable.

### Structural environment factors: psychosocial teleworking conditions and work fatigue

*Job demands* are job conditions that require sustained effort, for example workload and responsibilities, and are often the most important predictor of work fatigue/burnout within the COR and JDR models (Bakker et al., 2014). Studies have found links between high job demands/workload during the pandemic and work efficiency/productivity perceptions, or higher work engagement (Da et al., 2022). In these pandemic studies, links are mostly found when home environments had minimal distractions/interruptions and were work-conducive (Baert et al., 2020; Ipsen et al., 2020; YouGov, 2020). Presumably, in a pandemic context high job demand can also act as a motivational driving force under the right circumstances, as shown in pre-pandemic research (van den Broeck et al., 2010; Bakker and Sanz-Vergel, 2013). However, a different stream of pandemic research indicates that job demands have changed in nature and intensity due to new pandemic-specific job demands, such as teleworking-specific tasks (e.g., consecutive online meetings inhibiting breaks, Xiao et al., 2021; Syrek et al., 2022), disruptive teleworking management tasks (Chong et al., 2020) and telework never seeming to end (Wang et al., 2020). High levels of job demand during pandemic telework have been associated with increased perception of stress (Hayes et al., 2020), emotional work exhaustion/fatigue (Chong et al., 2020; Wang et al., 2020; Abdel Hadi et al., 2021), and burnout (Hayes et al., 2020), especially when adequate job resources were not in place (e.g., telework task support or leader support; Chong et al., 2020; Da et al., 2022) and when (retrospectively) compared to pre-pandemic work (Hayes et al., 2020).

*Job control* is defined as the perceived level of autonomy and influence workers have over when and how they work; e.g., autonomy in scheduling work, making decisions, and choosing working methods (Bakker and Demerouti, 2007). Pre-pandemic teleworking research indicates that teleworking is predominantly advantageous for job control, with teleworking enhancing perceived job control in terms of when and where work is done and how it fits around other aspects of life (Mann et al., 2000; Dambrin, 2004; Gajendran and Harrison, 2007; vander Elst et al., 2017). In pre-pandemic research, job control is consistently positively associated with wellbeing and negatively associated with indicators of burnout, including work fatigue (Fernet et al., 2004; Taris et al., 2005; Alarcon, 2011; Park et al., 2014; Frone and Tidwell, 2015). However, there is still little evidence on the salience of this association (job control-work fatigue) in the pandemic teleworking context as studies have not tested associations with a global operationalisation of work fatigue (emotional, mental, and physical, Frone and Tidwell, 2015) but with related constructs such as emotional fatigue (Chong et al., 2020; Wang et al., 2020), non-work-specific exhaustion (Meyer et al., 2021), and wellbeing (Straus et al., 2022). Furthermore, studies suggest that prior knowledge cannot be readily transferred as pandemic working poses new issues - such as daily COVID-19 task setbacks (Chong et al., 2020) - and/or intensified old issues, such as family-work-interference (Wang et al., 2020).

*Job change* captures how well any organisational change is managed and communicated (HSE Management Standards Approach, 2009). Organisational change, which may include changes to one’s own job, is associated with work fatigue/exhaustion and burnout (Dubois et al., 2013; Day et al., 2017). Good management of organisational or job change, e.g., high-quality supervisor, peer support, or providing training, is a resource that can help employees cope with change-related stress (Mitchell, 2018; European Commission, 2020) and therefore avoid work fatigue (Day et al., 2017; Guidetti et al., 2018). Since teleworkers often have reduced opportunities for support and feedback from colleagues (Sardeshmukh et al., 2012) they are at risk of negative outcomes arising from job change. This risk was accelerated by the fast organisational changes due to the pandemic (Amis and Janz, 2020); a large number of workers were suddenly primarily teleworking, a shift that organisations and employees were largely unprepared for with more than half of workers in European Union countries having had no prior experience with teleworking (European Commission, 2020; Hofmann et al., 2020; Kaushik and Guleria, 2020). Nonetheless, very few studies investigated the role of job change in the pandemic teleworking experience. Some longitudinal evidence suggests that being satisfied with organisational communication about COVID-19 related work changes is an important resource to protect wellbeing during pandemic teleworking (Straus et al., 2022). However, overall, the role of perceived job change management in work fatigue during pandemic teleworking has been rather neglected despite researchers having called for it (*cf.* Escudero-Castillo et al., 2021).

In terms of the structural environment, this study contributes to emerging evidence about pandemic telework by exploring relationships between job demands, job resources (job control and job change) and work fatigue during the first COVID-19 lockdowns in 2020. We propose that:

*H1*: Individuals reporting higher job demand, lower job control and poorer job change management during the COVID-19 lockdown will report greater work fatigue (emotional, mental, and physical) after controlling for all other predictors.

### Built environment factors: privacy fit, home-office characteristics, and work fatigue

*Work privacy fit*^1^ addresses the home office from a socio-spatial level to determine its adequacy to fulfil work privacy needs (Weber et al., 2021). Work privacy fit is a multidimensional conceptualisation and operationalisation of work privacy, which builds on Altman’s privacy regulation framework (1975) that is related to Person–Environment fit theory (Edwards et al., 1998). As such, work privacy is regarded as “a control process of input and output of information and social stimuli in the work environment. Workers attempt to regulate stimuli coming from their colleagues and output they make to their colleagues. Workers strive to achieve the best possible fit between their actual and desired levels of input and output” (Weber et al., 2021 p. 70). Four distinct dimensions of work privacy are considered: “distractions (regulation of indirect social stimuli/input), interruptions (regulation of direct social stimuli/input), task privacy (regulation of visual output) and conversation privacy (regulation of acoustical output)” (p. 70). For further detail on the conceptual underpinning of work privacy, please refer to Weber et al. (2021). Congruent with Person-Environment fit principles, it is possible to maximize work- and health-related outcomes if environmental characteristics can be organised in a way that supports individual privacy needs (Weber et al., 2021). Pre-pandemic research indicates that work privacy fit in an office context drastically shapes the work experience, as it is associated with various work-related (e.g., social conflict mitigation, Peters and Knoll, 2020, self-rated productivity, *cf.* Weber et al., 2021), and occupational health outcomes, such as work fatigue (*cf.* Weber et al., 2021). Most pandemic studies on privacy fit or privacy-related aspects (e.g., work and non-work distractions and interruptions) have explored the impact on work efficiency and performance perceptions (Ipsen et al., 2021; Leroy et al., 2021; Pfnür et al., 2021; Bergefurt et al., 2022; Weber et al., 2022; Park et al., 2023). Few studies have considered the relative effects of privacy fit on health and wellbeing. Those that do have concentrated on reduction of sleeping problems (explained through cognitive irritation, Wütschert et al., 2022) and musculoskeletal complaints (Wütschert et al., 2022) when privacy was given. Studies that focused on occupational health have only observed aspects that were related to poor privacy fit (different types of interruptions, *cf.* Leroy et al., 2021, distractions, Bergefurt et al., 2021). These studies indicated negative associations with overall stress (Bergefurt et al., 2021, 2022; see footnote 1), mood (Bergefurt et al., 2022), dimensions of burnout (incl. Emotional fatigue, Bergefurt et al., 2021; Leroy et al., 2021), and multiple aspects of mental health (Xiao et al., 2021). However, none of the studies distinctly assessed the relationship between all dimensions of privacy fit (distractions, interruptions, task and conversation privacy) and all dimensions of work fatigue (emotional, mental, physical).

In this study, we examined work privacy fit as a factor in the built environment and examined its relationship to work fatigue during lockdown teleworking. We propose that:

*H2*: Individuals reporting higher levels of work privacy fit will report lower levels of work fatigue (emotional, mental, and physical) during the COVID-19 lockdown after controlling for all other predictors.

*Home office characteristics as predictors of privacy fit:* Pandemic research indicates drastic differences in home office environments supporting or hindering privacy across samples. Some experienced privacy-related advantages, such as less distractions/interruptions, that were related to increases in concentration and productivity (Ipsen et al., 2020, 2021; Pfnür et al., 2021). Others reported problems with privacy, distraction, or interruptions since pandemic teleworking (Ipsen et al., 2020; Bergefurt et al., 2021; Ipsen et al., 2021; Leroy et al., 2021; Xiao et al., 2021; Bergefurt et al., 2022; Wütschert et al., 2022; Park et al., 2023). Considering the acknowledged impact of privacy fit on occupational health, likely predictors of privacy fit ought to be explored. Based on pre-pandemic (Marshall, 1972; Weber et al., 2021) and pandemic evidence, this study focuses on three key predictors of privacy fit: shared/unshared workspace, perceived noise levels, and crowding. Pandemic research indicates that unshared workspaces at home were associated with fewer non-work (family) interruptions or distractions (Leroy et al., 2021; Bergefurt et al., 2022; Bezak et al., 2022), perceived workspace suitability (Toivonen et al., 2022) or perceived performance loss (Puglisi et al., 2021). Similarly, perceived social density/crowding or number of people at home while teleworking was related to lack of privacy (Bezak et al., 2022; Park et al., 2023), disturbances (Baert et al., 2020) or perceived workspace suitability (Toivonen et al., 2022). As evident from pre-pandemic research, household size can make it difficult to regulate social interactions and achieve good privacy fit (Marshall, 1972), and the availability of an unshared room for work has been positioned as critical success factor for telework (Yap and Tng, 1990; Baruch and Nicholson, 1997). Further, perceived noise exposure was related to perceived home-office distractions (Bergefurt et al., 2022), workspace suitability, and perceived performance loss (Puglisi et al., 2021).

To further understand the impact of the built environment during lockdown teleworking, we examined how home office characteristics affected privacy fit. We propose that:

*H3*: Individuals reporting more noise, more crowding, and who work with others in the same room will report lower work privacy fit (emotional, mental, and physical) during the COVID-19 lockdown after controlling for all other predictors.

### Social environment factors: childcare responsibilities, privacy fit and work fatigue

Telework is promoted as a way to reduce work–family conflict because it allows flexibility of time management and reduces the need to commute between different locations (Sakamoto and Spinks, 2008). However, this flexibility can also have negative impacts on boundaries between professional and domestic spheres (Gajendran and Harrison, 2007). For women, family appears more likely to intrude into work time whereas for men telework may be seen as an opportunity to work for longer or in a more focused way (Sullivan and Lewis, 2001). Where work already conflicts with family demands, intensive teleworking from home has long been positioned to lead to interference between the two, ultimately increasing work fatigue (Golden, 2012). In the context of nursery and school closures as a result of COVID-19 lockdowns, many parents had to combine full-time work with childcare and education in their home environment. Emerging empirical evidence indicates this has resulted in reduction of work efficiency, high self-reported loneliness, anxiety, depression, psychological distress, and overall reduced physical and mental wellbeing among parents, especially among mothers (Cox and Abrams, 2020; Ipsen et al., 2021; Kerr et al., 2021; Meyer et al., 2021; Shockley et al., 2021; Xiao et al., 2021), and increased the risk for parental burnout^2^ (Kerr et al., 2021; Griffith, 2022; Woine et al., 2022). The presence of and number of children, work-home conflict, home-to-work interference, and home demand have also been associated with emotional fatigue (Wang et al., 2020; Abdel Hadi et al., 2021), job burnout (Barriga Medina et al., 2021; Da et al., 2022), non-work specific exhaustion (Meyer et al., 2021), and reduced physical and mental health (Xiao et al., 2021). These are made worse in combination with low levels of family-to-work facilitation (Da et al., 2022), low social support (Wang et al., 2020), low job control, and low partner support (Meyer et al., 2021).

In terms of the social environment, this study contributes to emerging evidence about pandemic telework by exploring the relationship between childcare and work fatigue during lockdown. We propose that:

*H4*: Childcare responsibilities will be positively related to work fatigue (emotional, mental, and physical) during the COVID-19 lockdown, after controlling for all other predictors.

*Privacy fit as a mediator:* a considerable number of studies indicate incremental links between some, or all, of the following variables: childcare responsibilities; privacy/distractions/interruptions; work-family/family-work interference/conflict; fatigue/wellbeing (Baert et al., 2020; Ipsen et al., 2020, 2021; Leroy et al., 2021; Xiao et al., 2021; Bergefurt et al., 2022; Syrek et al., 2022; Wütschert et al., 2022; Park et al., 2023). These links appeared particularly pronounced or are hypothesised to be, when the home environment did not cater for spatial separation (e.g., dedicated home-office) but forced individuals to share the work room with household members (Seva et al., 2021; Bezak et al., 2022). For example, Leroy et al. (2021) showed relationships between non-work responsibilities (childcare, family responsibilities and household), shared/unshared workspace at home, number of non-work interruptions, and emotional exhaustion. Childcare responsibilities predicted more interruptions whereas dedicated workspace predicted fewer interruptions; in turn, interruptions predicted emotional exhaustion. As such, we suggest that a significant amount of variance in the relationship of childcare on work fatigue is explained by privacy fit.

To further understand the impact of the social and built environment during lockdown teleworking, we therefore examined the potential for privacy fit to explain any relationship between childcare and work fatigue. We propose that:

*H5*: Privacy fit will mediate the relationship between childcare responsibilities and work fatigue (emotional, mental, and physical) during the COVID-19 lockdown after controlling for all other predictors.

### Control variables at the individual and structural levels: gender, age, and country

We included gender, age, and country as control variables, as research suggests they may influence experiences of pandemic teleworking. For example, women have reported to be experiencing more fragmented time whilst pandemic teleworking due to family-home interference (Leroy et al., 2021) and as such, gender-related differences in privacy fit are likely. Gender differences have also been reported in mental health whilst teleworking (Wang et al., 2020) and in pre-pandemic work fatigue research (Posig and Kickul, 2004). Regarding age, studies have indicated age-related differences in pandemic working experiences (Ipsen et al., 2021). Country variation has been observed in teleworking preparedness with regards to equipment and home office environments (European Commission, 2020) and there were also country differences in terms of strictness of social distancing measures and other restrictions during COVID-19 lockdowns.

## Methods

### Study design and procedure

An online, cross-sectional survey using the platform ‘Limesurvey’ was conducted with an opportunistic sample of workers, recruited mostly in three primary countries associated with the research group: Germany, Switzerland, and the United Kingdom. Participants were also recruited from other countries (Australia, Austria, Belgium, Canada, Czech Republic, Denmark, Finland, France, Greece, India, Italy, Japan, Luxembourg, Netherlands, Portugal, Turkey, and Zimbabwe). The survey was administered in English as a key measure (work privacy fit) was only available in English at the time. Given the particular limitations of conducting research during the pandemic, this also helped to reduce the procedural complexity of data collection.

The survey launched on April 10th 2020, when lockdown or strict social distancing measures had been in place between 18 and 26 days across the primary countries (commencing March 22nd in Germany, March 16th in Switzerland, and March 23rd in United Kingdom). Data were collected until May 2nd 2020. Participants were recruited *via* social media (Twitter) and email among the researchers’ extended networks of colleagues, friends, and family. Inclusion criteria were that during the previous 2 weeks of the COVID-19 lockdown (e.g., the 2 weeks prior to survey completion) participants: (a) were aged 18 years or older, (b) were working and (c) had primarily worked from home.

A subset of the data (*n* = 184) has been analysed and reported in a previous study, which examined the role of work privacy fit, job demand, job control, and job change in predicting future teleworking intentions (Weber et al., 2022).

### Participants and ethics

Ethical review and approval were not required for the study on human participants in accordance with local legislation and institutional requirements. Participants were given the option to particate voluntarily and were required to provide written informed consent if they agreed to be in the study. All survey data were anonymised to make it impossible to gather any identifying information. Data were shared only among the research team and all data were stored on a secure university server. Data collection procedures and data use were undertaken carefully so as to conform with the Swiss Federal Data Protection Act. As the COVID-19 lockdown was for some a stressful life-event, a debrief page was provided to participants. This was specific to each of the three primary countries and provided links to healthcare providers and other sources of available online support.

A total of 737 respondents participated, of which 258 were excluded in the first data cleaning step due to extensive missing data (i.e., no responses apart from demographics) or illogical responses (i.e., illogical text in text fields) suggesting non-valid submission of responses. This resulted in a sample of 479 respondents which included some missing data. A total of 99 cases had missing data among individual items of the study variables (job demand, job control, job change, privacy fit, crowding, noise and work fatigue). Little (1988)‘s Missing Completely at Random Test on all ordinally scaled study variables suggests it is unlikely for there to be systematically missing data (*χ*^2^ (67, *n* = 380) = 62.16, *p* = 0.65) and is congruent with listwise deletion. As a second data cleaning step, all cases with missing data on the study variables were deleted listwise resulting in a final sample of 380 respondents. In the final data cleaning step, all cases (*n* = 80) that indicated a ‘not applicable’ (N/A) response on job demand, job control and job change items were excluded from the analysis. The final data set comprises *n* = 300.

Participation by primary country was almost evenly distributed (United Kingdom, 34.0%; Switzerland 24.7%; Germany, 24.0); 17.4% of responses stemmed from ‘other countries’. The gender distribution among participants was uneven; almost two times more females (64.7%) than males (35.3%) took part. The majority of participants (88.0%) fell in the age groups 21-30 (20.0%), 31-40 (44.0%), and 41-50 (24.0%). Approximately a third of the sample (30.3%) reported to have childcare responsibilities (caretaking or/and home schooling) while pandemic teleworking. However, 32.0% reported that between one to four children under the age of 15 years^3^ were present at home. As such, not all participants who had children present when teleworking had caretaking responsibility; this discrepancy was only present in responses from males.

Regarding teleworking arrangements before the pandemic, 43.0% had teleworked from home before, on average at a ratio of 33.3% per week. During the pandemic, participants worked on average 35.57 h (*SD* = 13.22) per week at home, which was for 42.0% of participants about the same as before the pandemic (29.2% reported less home-working than before; 28.8% reported more than before). The majority of participants (64.4%) stated that their working pattern (e.g., later start and end, dispersed working hours) has changed during pandemic teleworking.

Regarding participants’ home office environment, 18.3% had a dedicated home office, 57.6% worked either on reallocated furniture (e.g., dining table, 31.3%) or a dedicated desk in a room also used for other purposes (26.3%). Overall, the median number of people present at home when home working was 3 (range 1-7). If others were also working from home, 71.6% worked in separate rooms in relation to the participants; 28.4% shared their room (15.4%) or their work area (e.g., desk, 13%). Detailed participant demographics and home office information are provided in Table 1.

**Table 1:** Demographic details of the sample.

Characteristic		Count	Percentage
**Country**			
	United Kingdom	102	34.0
	Switzerland	74	24.7
	Germany	72	24.0
	Other*	52	17.3
**Gender**			
	Male	106	35.3
	Female	194	64.7
**Age**			
	16-20 years	0	0.0
	21-30 years	60	20.0
	31-40 years	132	44.0
	41-50 years	72	24.0
	51-60 years	31	10.3
	61-70 years	5	1.7
**No. of children <15 years**			
	0	204	68.0
	1	46	15.3
	2	41	13.7
	3	8	2.7
	4	1	0.3
**Childcare**			
	Yes	91	30.3
	No	209	69.7
**Own work location**			
	Home office	55	18.3
	Room used as office	57	19.0
	Dedicated desk	79	26.3
	Reallocated furniture	94	31.3
	Other	15	5.0
**Others work location**			
	Separate rooms	204	68.0
	Same room separate area	44	14.7
	Same room and same area	37	12.3
	NA – no other teleworkers.	15	5.0

*N* = 300. *Other countries include: Australia, Austria, Belgium, Canada, Czech Republic, Denmark, Finland, France, Greece, India, Italy, Japan, Luxembourg, Netherlands, Portugal, Turkey, Zimbabwe.

### Measures

Measures are described below. Descriptive statistics and correlations are provided in Table 2.

**Table 2:** Means, standard deviations, and correlations between the study variables.

Variable		*M / %*	*SD*	1	2	3	4	5	6	7	8	9	10	11	12	13	14	15	16	17	18	19	20
1	Age	3.30	0.95	–																			
2	Female	64.7%	–	−0.06	–																		
3	United Kingdom	34%	–	0.06	0.17**	–																	
4	Switzerland	24.7%	–	−0.02	−0.24***	−0.86***	–																
5	Germany	24%	–	0.01	−0.07	−0.85***	−0.79***	–															
6	Childcare - Yes	30.3%	–	0.21***	0.01	0.08	−0.27***	0.18**	–														
7	Home office	18.3%	–	0.21***	0.02	0.04	0.05*	−0.01	0.05	–													
8	Room used as office	19%	–	0.19***	−0.16**	−0.12*	0.09	0.16**	0.02	−0.68***	–												
9	Dedicated desk	26.3%	–	−0.16**	−0.03	0.14*	−0.18***	−0.10	0.12*	−0.75***	−0.75***	–											
10	Reallocat. furniture	31.3%	–	−0.12*	0.08	−0.13*	0.02	−0.05	−0.18**	−0.79***	−0.79***	−0.85***	–										
11	OWL: Sep. rooms	68%	–	0.12*	−0.07	0.04	0.01	0.06	−0.02	0.17**	0.34***	−0.08	−0.30***	–									
12	OWL: Same room sep. area	14.7%	–	−0.08	0.17	−0.16**	0.01	0.11	0.07	0.10	−0.20***	−0.03	0.13*	−0.94***	–								
13	OWL: Same room & area	12.3%	–	−0.26***	−0.08	0.11	−0.06	0.16**	0.08	−0.37***	−0.20***	0.06	0.27***	−0.93***	−0.53***	–							
14	Job demand	2.47	0.90	0.08	0.15**	0.19***	−0.19***	−0.14*	0.11	0.12*	−0.01	−0.05	0.02	0.01	−0.07	−0.26***	–						
15	Job control	3.91	0.72	0.12*	0.01	−0.03	0.17***	−0.06	−0.04	−0.16**	0.08	0.08	−0.11	0.03	0.01	0.10	−0.27***	–					
16	Job change	3.50	0.91	0.02	−0.04	0.04	0.23***	−0.12*	−0.08	0.02	−0.02	0.07	−0.10	0.16**	−0.03	−0.16	−0.38**	0.36***	–				
17	Work privacy fit	2.88	3.95	0.01	−0.06	−0.18**	0.20***	0.06	−0.57***	0.28***	0.02	−0.07	−0.18**	0.18**	−0.13*	−0.28***	−0.17**	0.14*	0.18**	–			
18	Noise	2.50	1.29	−0.09	0.08	0.12*	−0.11	−0.16**	0.46***	−0.30***	0.01	0.06	0.20***	−0.21***	0.01	0.41***	0.16**	−0.09	−0.09	−0.55***	–		
19	Crowding	2.35	1.18	−0.02	−0.02	0.06	−0.16**	−0.04	0.46***	−0.21***	0.03	0.05	0.14	−0.19***	0.10	0.29***	0.24***	−0.20***	−0.12*	−0.50***	0.58***	–	
20	Work fatigue	2.60	1.02	0.01	0.25***	0.27***	−0.33***	−0.15**	0.20***	−0.08	0.01	0.07	0.05	−0.08	−0.07	0.12*	0.41***	−0.13*	−0.21***	−0.33***	0.35***	0.31***	–

*N* = 300. *p < 0.05, **p < 0.01, ***p < 0.001 (2-tailed). % represents the percentage of the value of “1” where dummy coding was used. OWL = Others’ work location. Reference dummy variables Country: Other Countries; Gender: Male; Own work location: Other; Others’ work location/OWL: NA – no other teleworkers; Childcare: No childcare.

#### Demographics (including control variables)

Data were collected on age, gender, country of residence during previous 2 weeks of lockdown, number of children aged under 15 years, and childcare responsibilities (additional information below). Information about teleworking was also collected, including teleworking start date, prior teleworking arrangements and the percentage of time teleworking pre-lockdown, hours per week worked, whether they had worked more or less since the pandemic, and if they had changed their work pattern during pandemic teleworking. Country and gender categories were dummy coded with ‘other countries’ and ‘male’ being the referent.

#### Structural environment factors: psychosocial teleworking conditions (job demand, job control, and job change)

*Job demand*, *job control*, and *job change* were assessed by the short version of the Health and Safety Executive (HSE) indicator tool (Edwards and Webster, 2012). The dimensions job demand (e.g., ‘I had unachievable deadlines’) and job control (e.g., ‘I had a say in my own work speed’) were each measured by four items. Job change was measured with three items (e.g., ‘Staff were always consulted about change at work’). Items were measured on a 5-point Likert scale, ranging from 1 (never) to 5 (always). The answer option N/A was added to account for those participants who were self-employed. N/A responses were discounted for the analysis. Internal consistency for all three dimensions was acceptable (α_jd_ = 0.80; α_jc_ = 0.73; α_jch_ = 0.73). Mean composite scores were calculated. High scores reflect high levels of job demand, job control, and job change.

#### Built environment factors: home office characteristics (crowding, noise, and workplace type), and work privacy fit

*Perceived noise* and *crowding* were measured with one item each (Marshall, 1972). Participants were asked to rate if the home office ‘felt crowded’, and if ‘it was noisy (inside the flat/house or outside)’. Items were measured on a 5-point Likert scale, ranging from 1 (strongly disagree) to 5 (strongly agree). High scores reflect high levels of perceived noise and crowding.

The *own work location* in the house/flat over the past 2 weeks was assessed with five categories (a dedicated home office; a room that is sometimes is used as an office; a dedicated desk in a room; a reallocated furniture; e.g., dining table); and other (Toivonen et al., 2022). The first four categories were dummy coded, with ‘other’ being the referent.

*Other home-workers’ primary work location* in relation to the participant was assessed with three categories (separate rooms; same room but separate area; same room and same area; e.g., shared desk). A fourth category was included (NA - no other teleworkers), to reflect participants who were not sharing their home with other home-workers. As with the previous dummy coding, the first three categories were dummy coded with ‘NA - no other teleworkers’ being the referent.

*Work privacy fit* was measured using a simplified version of Weber (2019) Privacy at Work (PAW) inventory. Participants rated their satisfaction with the level of privacy they experience at work based on the importance of four separate dimensions of privacy assessment: (1) conversation privacy / working without being overheard, (2) task privacy / working without being overseen (being watched over by others), (3) working without being interrupted, and (4) working without distractions. Explicitly, the items for satisfaction were: (1) ‘I was satisfied with the time I could work without being overheard’; ‘I had the opportunity to work without others listening into my work or non-work related conversations when I wanted to’ (2) ‘I was satisfied with the time I could work without being overseen’; ‘I had the opportunity to work without others seeing me or my work when I wanted to’. (3) ‘I was satisfied with the time I could work without being interrupted’; ‘I had the opportunity to work without engaging with anyone in my home when I wanted to’. (4) ‘I was satisfied with the time I could work without visual and acoustical distractions’; ‘I had the opportunity to work in a quiet and visually calm environment in my home when I wanted to’. Items were measured on a 5-point Likert scale, ranging from -2 (strongly disagree) to 2 (strongly agree). Internal consistency for privacy satisfaction and privacy importance was adequate (α_ps_ = 0.83; α_pi_ = 0.74). A composite score to reflect relative privacy fit was created by weighting privacy satisfaction ratings with privacy importance ratings using multiplication (*cf.* Lindner et al., 2016). High scores reflect high levels of work privacy fit.

#### Social environment factors: childcare responsibilities

*Childcare responsibilities* were measured using a single item as part of the demographics section of the survey. Participants were asked whether they had to look after their children (e.g., caretaking, homeschooling) in addition to working from home; they could answer ‘yes’ or ‘no. These categories were dummy coded with ‘no childcare’ being the referent.

#### Work fatigue

*Work fatigue* was assessed using an 18-item measure by Frone and Tidwell (2015) on a five-point Likert scale ranging from (1) never to (5) every day. This three-dimensional work fatigue inventory takes into account three different resource-specific types of fatigue at work: emotional fatigue, mental fatigue, and physical fatigue (e.g., ‘how often did you feel emotionally/mentally/physically exhausted at the end of the workday’). An overall fatigue mean composite score across all dimensions was calculated. The wording was amended to suit the study by using a reference frame of the last 2 weeks as opposed to the original reference frame of 12 months. Internal consistency was excellent (α = 0.97). High scores reflect high levels of work fatigue.

### Data analysis

The statistical software package R version 4.2.2 (R Core Team, 2020) was used to compute descriptive statistics and correlation matrices. We used Pearson correlations where both variables were continuous, tetrachoric correlations between two dichotomous variables, and point-biserial correlations between a dichotomous and continuous variable. The lavaan package (Rosseel, 2012) and semTools package (Jorgensen et al., 2021) in R were used to test the path model and indirect effects using the MLM estimator as per Hypotheses 1-5. The default confidence intervals from the lavaan package were used.

## Results

**Figure 2:**
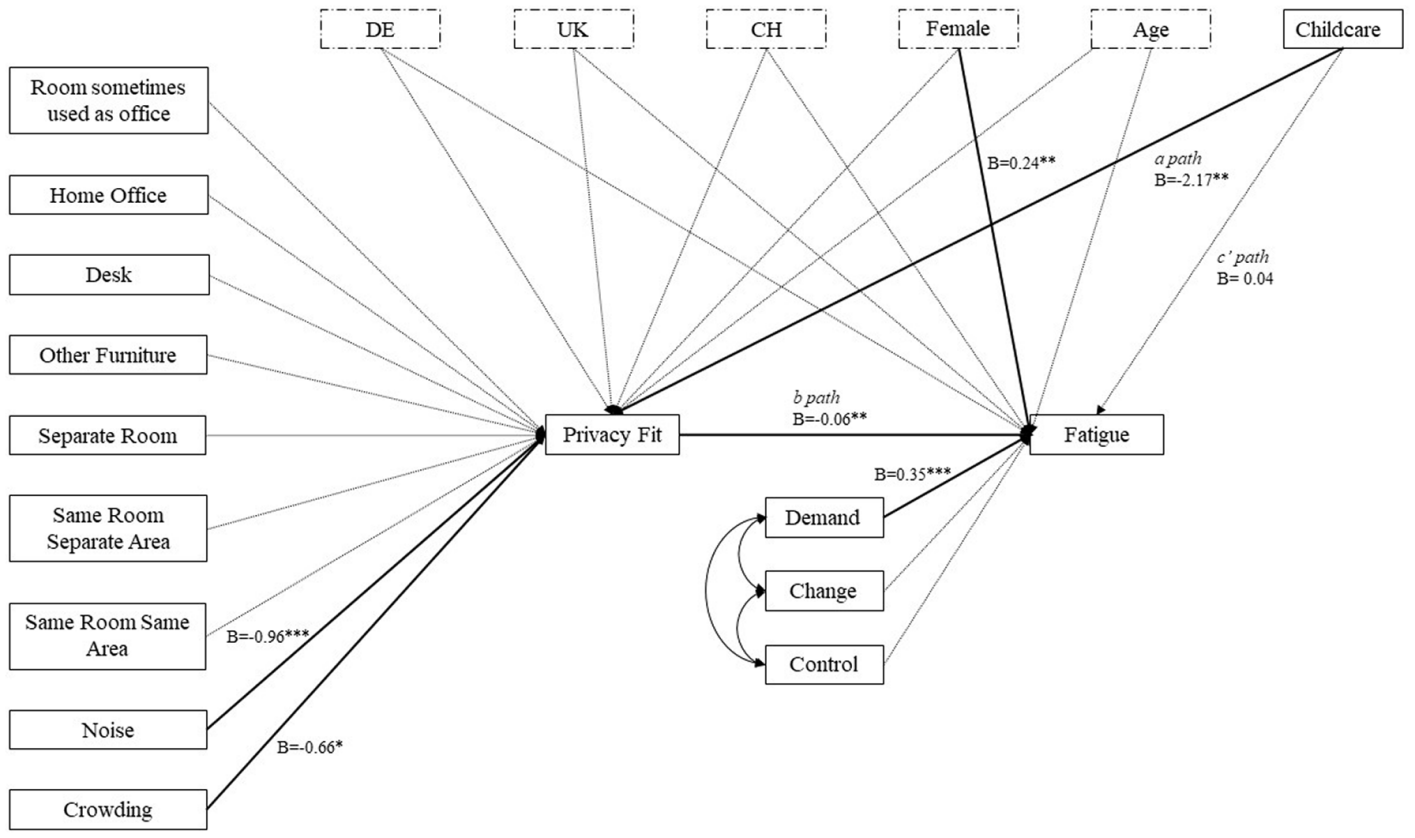
Path analysis results. Control variables are indicated by dashed lines. Insignificant relationsips are indicated by grey lines. Unstandarized coefficients are reported. *p < 0.05; **p < 0.01; ***p < 0.001; *n* = 300. Referent dummy variables country: other countries; gender: male; own work location: other; others’ work location: NA – no other teleworkers; childcare: no childcare.

The proposed model had a good fit with their robust estimators (Hu and Bentler, 1998; Byrne, 2013) (RMSEA = 0.03; CFI = 0.92; TLI = 0.89; SRMR = 0.05), with chi-square (*χ*^2^ = 70.90, 57, *p* = 0.10) not being significant. An overview of the final model, with details of direct relationships between variables is shown in Figure 2. Of the control variables (age, gender, country), only gender predicted work fatigue; females were more likely to report greater levels of work fatigue.

*H1* was partially supported, as higher job demands (B = 0.35; 95% CI 0.20 to 0.49) predicted higher levels of work fatigue. However, neither job control nor job change predicted work fatigue as we had hypothesised. Congruent with *H2*, individuals’ levels of work privacy (B = −0.06; 95% CI −0.09 to −0.03) negatively predicted their levels of work fatigue during the COVID-19 lockdown; those who reported poorer work privacy experienced greater work fatigue.

Figure 2 and Table 3 show partial support for *H3*, where noise (B = −0.96; 95% −1.33 to −0.47) and crowding (B = −0.66; 95% CI −1.20 to −0.13) both negatively predicted work privacy fit during COVID-19 lockdown. Noisier and more crowded home-working spaces were associated with poorer work privacy fit. Contrary to our hypothesis, however, if and how workspace was shared during lockdown teleworking did not predict privacy fit.

**Table 3:** Path analysis results including mediation.

Predictor	Work privacy fit		Work Fatigue	
	B (SE)	*z*	B (SE)	*z*
United Kingdom	−0.73 (1.96)	−0.37	−0.02 (0.40)	−0.04
Switzerland	0.17 (1.78)	0.10	−0.45 (0.35)	−1.31
Germany	0.02 (2.00)	0.01	−0.32 (0.39)	−0.81
Age	−0.13 (0.39)	−0.34	−0.03 (0.07)	−0.35
Female	−0.11 (0.47)	−0.23	0.24 (0.11)	2.18**
Childcare (a path)	−2.17 (0.84)	−2.85**	(c’ path) 0.04 (0.17)	0.24
Noise	−0.96 (0.25)	−3.74***		
Crowding	−0.66 (0.27)	−2.41*		
Home office	1.06 (2.38)	0.36		
Room used as office	0.21 (2.62)	0.07		
Dedicated desk	−0.02 (2.44)	−0.01		
Reallocat. furniture	−0.52 (2.46)	−0.17		
OWL: Sep. rooms	−1.29 (1.87)	−0.69		
OWL: Same room sep. area	−2.10 (2.19)	−0.96		
OWL: Same room & area	−1.75 (2.18)	−0.80		
**Predictor**				
Job demand			0.35 (0.07)	4.65***
Job control			0.03 (0.09)	0.38
Job change			−0.04 (0.07)	−0.63
Work privacy fit (b path)			−0.06 (0.02)	−3.54**

*N* = 300. *p < 0.05, **p < 0.01, ***p < 0.001 (2-tailed). OWL = Others’ work location. Referent dummy variables Country: Other Countries; Gender: Male; Own work location: Other; Others’ work location: NA – no other teleworkers; Childcare: No childcare. Model fit: *χ*^2^ = 70.90, 57, *p* = 0.10; RMSEA = 0.03; CFI = 0.92; TLI = 0.89; SRMR = 0.05.

No support was found for *H4*, as childcare responsibilities did not directly predict work fatigue; however, in line with *H5*, an indirect relationship was observed between childcare responsibilities and work fatigue with work privacy fit being a significant mediator (B = 0.13; 95% CI 0.02 to 0.23). Those with childcare responsibilities experienced greater work fatigue, as a result of experiencing poorer work privacy fit.

A *post-hoc* power analysis showed that a sample size of *n* = 300 yields a power of 73% to reject a wrong model (with df = 175) with an amount of misspecification corresponding to RMSEA = 0.03 on alpha = 0.05. This is below the recommended threshold of 80% suggesting a slightly underpowered model. However, given the concern around *post-hoc* power testing (Althouse, 2021), the primary implication is recognizing the need for a larger study sample in future studies and the importance of power testing in the study design period.

## Discussion

This cross-sectional study examined predictors of work fatigue during home-based teleworking in the first COVID-19 lockdown, by examining the influence of various factors across different levels of a social-ecological model of occupational health (Stokols, 1996; Sallis and Owen, 2015; Munir et al., 2021). Work fatigue was influenced by factors from the structural/psychosocial, built, and social environmental levels, and at the individual level (gender). On average, our sample was (with a slightly positively skewed distribution) only moderately work fatigued; they experienced work fatigue around once a week in the 2 weeks prior to participation. Women were more fatigued than men, which confirms previous pandemic and pre-pandemic evidence (Posig and Kickul, 2004; Wang et al., 2020). However, these results should be interpreted with caution due to study limitations (e.g., the modest sample size of individuals that had childcare responsibilities, cross-sectional mediation analysis) hindering the estimation of robust effects. Furthermore, we recognise that, despite the challenges associated with the sudden transition to teleworking, those workers who were able to predominantly or exclusively telework during the early stages of the COVID-19 pandemic may well have experienced lower levels of job insecurity, psychological distress, physical health issues, and fear of the SARS-CoV-2 virus than workers who continued working on-site throughout lockdowns and other restrictions (Rudolph et al., 2021; Sischka et al., 2022).

With regards to work fatigue predictors, we found two predictors in our dataset. The first and strongest predictor - job demands - was at the structural/psychosocial level. Other variables at this level (job control and job change management) were not significantly associated with work fatigue in our sample. The second strongest predictor - work privacy fit - was at the built environment level. Also at the built environment level, the variables noise and crowding perceptions were significant predictors of privacy fit but shared/unshared workspace was not associated with work fatigue. The social environment level predictor - childcare - did not predict work fatigue directly. Instead, childcare responsibilities had an indirect effect on the likelihood of teleworkers experiencing work fatigue, and this association was positively mediated by privacy fit. To provide an in-depth discussion of the associations observed, each result is discussed by its socio-ecological level.

### Structural environment factors: psychosocial teleworking conditions

Our results show that job demand levels were low and job control and job change were rather high in our sample. We observed a positive association between job demands and work fatigue, which is theoretically consistent (Bakker et al., 2014), and reflects findings from other pandemic studies indicating work fatigue-related effects (Chong et al., 2020; Hayes et al., 2020; Wang et al., 2020; Abdel Hadi et al., 2021). However, overall, pandemic studies have indicated that the experience of job demands and associated impacts varies drastically across samples. Some studies identified additional, pandemic-specific job demands (e.g., all-day conference calls) impacting teleworkers’ mental health (Chong et al., 2020; Xiao et al., 2021; Syrek et al., 2022). In contrast, other teleworkers, mostly in work-conducive built environment conditions, experienced high demands alongside a sense of higher productivity and engagement (Da et al., 2022). This heterogenic picture is at least partially related to differences in sectors, jobs and related teleworking readiness. However, the varying experiences are also likely related to differences in the home environment and social life domain. For example, if teleworkers experience low levels of socio-environmental stress (low levels of crowding, adequate/non-shared home office space, low noise levels); if they can regulate social contact at home; and if they do not have children or care responsibilities, they are more likely to have the ability to recover from the strain of high job demand. In this scenario, high levels of job demand can act as a motivational factor (van den Broeck et al., 2010; Bakker and Sanz-Vergel, 2013). Apart from permanent contextual factors (such as the built environment), varying levels and results of job demands across pandemic studies should also be interpreted in the context of when data were collected. Workload levels were dynamic during COVID-19, following a U-shape time trend; longitudinal data shows a dip in workload in March 2020 when pandemic lockdowns first occurred, followed by a steady increase from April to May 2020 (Syrek et al., 2022); our results from April 2020 could fit this pattern. It seems likely that once pandemic-related changes decreased and practical problems at the start of pandemic teleworking (e.g., lack of hardware and software) were resolved, people resumed their work and projects started again, leading to rising workload in May (Syrek et al., 2022).

The high level of job control also aligns with levels and time trends from other longitudinal studies. For example, Syrek et al. (2022) suggest control increased from February 2020 onwards. It appears that workers experienced new levels of control and responsibility over their own work time when switching to pandemic telework. However, we failed to detect any effects of job resource variables (job control and job change management) on work fatigue. This could be explained by small effect sizes and underpowered tests, as job resources are acknowledged to be weaker predictors of work fatigue (Bakker et al., 2014). Other pandemic studies could not detect known mitigation effects of job control (Gajendran and Harrison, 2007) on the relationship between work-family-interference and mental health (Wang et al., 2020); work-family-interference was positioned as too extreme to be mitigable. Similarly, the undetected effects of job control, our test for a job change-work fatigue association might have been underpowered. This notion is supported by pandemic evidence indicating that job change takes a subordinate role in explaining wellbeing-related phenomena, such as work engagement/vigour, when compared to other psychosocial job aspects such as job control or relationships during the pandemic (Wontorczyk and Rożnowski, 2022). Further, our assessment (HSE management standards) might have had more validity if we had adapted items and made the link to COVID-19-related job change management more apparent; we employed the standard items of the assessment. In fact, other studies that made the link explicit indicated wellbeing effects of good communication regarding COVID-19-related job changes (Straus et al., 2022). Overall, this suggests the relationship between job demands and resources are complex during pandemic teleworking and warrants a systematic analysis of pandemic evidence by considering data collection timepoints.

### Built environment factors: privacy fit and home office characteristics

On average our sample had positive privacy fit scores with a slightly negatively skewed distribution, which means that many were able to meet their work privacy needs at home. Those that had good privacy fit in terms of distractions, interruptions, task, and conversation privacy had significantly lower work fatigue levels. This lockdown-specific result is unsurprising given the body of evidence regarding the health effects of unsuccessful spatial regulation of social interaction (e.g., work on crowding and privacy; see Evans and Cohen, 1987; Gatersleben and Griffin, 2017). Therefore, our study supports previous hints at privacy fit-exhaustion associations that used elements of the privacy concept (distractions or interruptions; Leroy et al., 2021) and complements emerging pandemic evidence of privacy’s role in mental and physical health issues (e.g., sleeping problems and MSK pain, Wütschert et al., 2021, 2022). Furthermore, this result adds to the substantive body of evidence on the link between stress/fatigue/exhaustion, anthropomorphic noise and interruptions (Evans and Johnson, 2000; Jahncke et al., 2011; Kerr et al., 2020) from prior to the pandemic. Noise from other people and interruptions (unsuccessful input controls) represent two of four dimensions of the work privacy fit conceptualisation. However, work privacy fit, as tested here, also considers output controls, specifically task and conversation privacy. Therefore, the identified effect of work privacy fit on work fatigue broadens our understanding of social and environmental stressors and their impact on work fatigue.

As such, our study adds to growing evidence that providing workers with the ability to regulate social interactions (e.g., opportunities to retreat) can influence occupational health.

With regards to work privacy fit predictors, our results only identified noise and crowding perception to be significant, whereas the type of workspace (e.g., dedicated home office) or the type of sharing in the workspace (e.g., same room, different area) were not significant. Our study variables had each been identified in prior studies to relate to aspects of privacy, such as disturbances (Baert et al., 2020; Bergefurt et al., 2022; Bezak et al., 2022; Park et al., 2023). Pandemic research also indicates direct relationships – not *via* privacy – between these home office characteristics and health/wellbeing, such as psychological distress (e.g., noise: Kracht et al., 2021; Xiao et al., 2021, unshared workspace: Xiao et al., 2021, number of people/crowding: Kracht et al., 2021; Fornara et al., 2022; Park et al., 2023). This is not surprising since noise and crowding are acknowledged socio-environmental stressors which can have various work-relevant consequences on cognition (performance), behaviour (reduced helping behaviour), and affect (tension, anxiety, stress), and can pose a risk to health (*cf.* Evans and Cohen, 1987), which is similar to the attributes of privacy. However, the lack of any observed effects regarding the “objective” predictors in our study (if and how a workplace is shared) might be explained by underpowered test statistics, the fact that almost 72% of participants were able to work in a separate room (if others present), and possibly too much shared variance between the objective characteristics of the environment and the perceptions of noise and crowding (appraisal of the environment). Further, from a theoretical perspective, it ought to be mentioned that certain privacy conceptualisations treat crowding perceptions as an outcome of poor privacy (Altman, 1975) whereas others treat it as a predictor (Marshall, 1972). Indisputably, there is a significant overlap between privacy and crowding which are both transactional socio-environmental appraisals of the environmental condition. Thus, both relationships could be true depending on the underlying conceptualisations of crowding and privacy used.

In conjunction with other pandemic evidence, this study suggests that the possibility for withdrawal from crowded household situations can most likely help employees to protect their energy depletion, recovery process, and work focus. Qualitative accounts of other pandemic studies found that common rooms, such as the kitchen and living room, were permanently used by many as an alternative workspace, which clearly does not provide adequate withdrawal possibilities (Seva et al., 2021; Bezak et al., 2022). Indeed, the availability of a private room for work and a workplace that is work-conducive have been positioned as critical success factors for telework (Yap and Tng, 1990; Baruch and Nicholson, 1997).

Beyond obvious predictors of privacy, such as a private room, privacy appraisal can also be influenced by more nuanced aspects in the environment. Those can include personalization of spaces (Wells, 2000; Wells and Thelen, 2002; Laurence et al., 2013) and other appropriation behaviours (Vischer, 2008; Fonner and Stache, 2012; Wohlers and Hertel, 2017). By appropriating a space, (tele)workers change the meaning of a space according to their interests and “claim” the space (Vischer, 2008; Wapshott and Mallett, 2012). Space-claiming creates territories/boundaries of social and environmental control (*cf.* Vischer, 2008) which in turn is an acknowledged moderator for socio-environmental and environmental stress (Evans and Cohen, 1987; Lee and Brand, 2005; Lee and Brand, 2010; Wapshott and Mallett, 2012). This is exemplified by office studies showing moderation effects of personalization on perceived privacy and emotional fatigue (e.g., Laurence et al., 2013). Overall, privacy fit appraisal appears to be less related to the actual design of work environments and to depend more on psychological factors, such as control (Vischer, 2008).

### Social factor: childcare and its link to privacy fit and work fatigue

In our sample, approximately 30% reported having childcare responsibilities while teleworking. However, caring for children had an indirect effect on work fatigue in our study, whereas other pandemic studies identified direct effects (Wang et al., 2020; Abdel Hadi et al., 2021; Barriga Medina et al., 2021; Da et al., 2022). However, we found a positive indirect effect of childcare responsibilities on work fatigue *through* privacy fit, i.e., having childcare responsibilities negatively impacted privacy fit in homeworking spaces, which resulted in increased work fatigue. Given that privacy fit appraisal appears to be highly related to environmental control (Vischer, 2008), it is likely that teleworkers with childcare responsibilities not only experience more privacy violations but foremost feel less in control over their physical environment and social regulation possibilities. Further, we found that work fatigue was particularly pronounced in women.

Overall, this corresponds with other pandemic evidence about people with children at home while home-working; those with a caretaking role experienced more fragmented time with more interruptions, and in turn, were more exhausted (Leroy et al., 2021), especially when family/partner support and family-to-work facilitation was lacking (Wang et al., 2020; Meyer et al., 2021; Da et al., 2022). In this regard, qualitative pandemic evidence provided rich insights into pandemic workers’ lives (Bezak et al., 2022). They have portrayed workers who live with children as having no opportunity to withdraw due to space-sharing during working hours or switching work locations in the home. For example, during family dinner time when the dining table was used as workspace. Finding places that are quiet and free of clutter has been described as difficult for those with small children (Bezak et al., 2022). Further, echoing prior pandemic evidence (Wang et al., 2020; Meyer et al., 2021; Shockley et al., 2021; Da et al., 2022), in the present study, women appeared particularly burdened during the pandemic with pre-pandemic acknowledged differences of home-work intrusion and associated fatigue (Sullivan and Lewis, 2001; Posig and Kickul, 2004) intensifying during pandemic conditions. An illustrative example is provided by Meyer et al. (2021) study which indicated a curvilinear relationship between pandemic duration and non-work specific exhaustion in women. Exhaustion intensified during the beginning of the pandemic when childcare was not available, whereas exhaustion reduced when lockdown measures were eased. Partner support lessened the effect. In contrast, the exhaustion of men who worked from home and/or did not take care of children was minimally affected by the pandemic. Our findings confirm other pandemic evidence regarding gender disparities in psychological well-being amid the present pandemic. Overall, these results indicate the important role of social regulation in protecting mental health, especially in exceptional situations, such as when work and childcare have to be combined during the pandemic. Further, results indicate a persistence in gender differences in home-work interference and its impact on women’s exhaustion, calling for governmental and organisational support to address this issue long-term (e.g., government support, flexible working arrangements or necessary technology, *cf.* Meyer et al., 2021).

### Limitations

The present research is subject to several limitations. The first pertains to representativeness which is undermined by using convenience sampling and possible participation/self-selection bias, as only those workers with the capacity to be part of the study or who were dissatisfied with pandemic telework might have participated. As the lockdown posed new and intense challenges, it is possible that study participation was not possible for those struggling the most. Hence, our study might underestimate the negative impact of teleworking during lockdown.

Further, the sample is not representative for the entire teleworking population in the primary countries (Switzerland, Germany, and United Kingdom); neither is the sample representative across any specific occupational groups or sectors. The study registration was public, therefore anyone interested was able to participate which likely brings a broad distribution and diversity of occupational sectors and roles. However, owing to the rapid onset of the pandemic and our attempt to start data collection quickly, we recognise that we had overlooked to collect more occupational specific data such as type of sector, occupation, job role, or tasks. This information would be advantageous since across these factors, workers may differ in their experiences of teleworking, alongside their teleworking infrastructure and home office setup before (European Commission, 2020) and during the pandemic (Ipsen et al., 2020). Since countries worldwide introduced different containment measures during the crisis, it is likely that occupations are differently affected by the coronavirus pandemic and the results should be interpreted against this background. Thus, the impact of the forced transition to telework observed in our data may be more pronounced in certain occupations that are less accustomed to teleworking. Additionally, this could be further pronounced in countries with lower levels of digitalisation or in countries with less developed teleworking initiatives than those primarily observed in this study.

The second limitation pertains to the cross-sectional design of the study, which examined variables at a single moment in time, prevents causal inferences and is susceptible to common method bias (Podsakoff et al., 2003). However, given the rapid onset of the pandemic and the fast-moving pace of lockdowns being implemented early in 2020, we had limitations in terms of time and resource to plan and conduct a study with more advanced study design. As emerging pandemic research shows Straus et al. (2022) and Syrek et al. (2022), it proves valuable to observe the relationships between psychosocial, social, and occupational health variables longitudinally as the pandemic teleworking context is dynamic in which organisations and individuals adapt to the unprecedented changes to working life. Further, although testing the mediation effect with a cross-sectional sample is appropriate (Hayes, 2018), it still has significant limitations. Cross-sectional mediation analyses carry the risk of misrepresentation of psychological processes and ambiguity of the direction of the effect; longitudinal mediation models provide better representations of mediation processes (O’Laughlin et al., 2018). In addition, the low sample size has power implications, particularly as some key dummy variables (e.g., childcare, work location) were low in frequency. Therefore, the potential causal relationships identified in this study should be interpreted with caution and examined further using longitudinal designs and with larger sample sizes. In addition, relative importance analysis (Nimon and Oswald, 2013) could be used in future studies to test for the relative importance of each predictor in relation to the presence or absence of other predictors.

The third limitation pertains to the socio-ecological framework. It provides a useful lens through which to investigate the relationship between health outcomes and individual, social, environmental, and work factors, but it is possible that specific factors on each of these levels were unduly represented in our study. For this reason, further research that adopts a fuller reflection of all levels is warranted. This could explore data that have not been present in research to date, relating to, e.g., sectors, occupations, company size, self-employment, job tasks or job roles. It is predicted that when these factors are acknowledged, workers’ teleworking infrastructure may differ, alongside workers’ experiences of teleworking. Additionally, wider aspects of the social environment were not recorded in this study beyond a focus on family commitments. Study of the wider social environment could include support systems from co-workers, managers (Chong et al., 2020), and family (Meyer et al., 2021; Da et al., 2022). Furthermore, on the individual level, individuals’ traits and abilities, such as introversion/extroversion or sensory sensitivity, could be linked to differences in work fatigue levels as research has shown that people who are introverted or sensory sensitive are more quickly aroused and disturbed by environmental and socio-environmental stressors (for a summary, see Weber et al., 2021). Further studies could examine these factors in more detail and identify suitable traits for intervention targets. A full investigation of the context levels was beyond the scope of this study.

Lastly, since a considerable amount of cross-sectional pandemic research (including our own previous work, Weber et al., 2022) did not discuss the context of time in relation to data collection within the pandemic (e.g., beginning, end of, or after lockdown), it was difficult to position our findings within the current pandemic literature. As evident from longitudinal studies, demands and resources were highly dynamic (Straus et al., 2022) with nurseries and schools closing and re-opening (with vast country differences), and some teleworkers and organisations appeared to have achieved better management of the multiple demands on various context levels (job, environment, social; Straus et al., 2022; Syrek et al., 2022). As such, we hope that future pandemic research can further contextualise the aspect of time when presenting their findings.

## Conclusion

Our study offers insight into the impact of the first lockdown of the COVID-19 pandemic on employees’ occupational health. In line with existing knowledge, the psychosocial factor job demand was the strongest predictor of work fatigue. Our study also underlines the emerging importance of privacy fit and its predictors in the home office environment as well as its influence on one’s likelihood to experience work fatigue. The results also indicate that women were more fatigued than men and that childcare responsibilities became problematic when optimal privacy fit was not provided. Further, it shows the different capabilities of teleworkers for post-pandemic teleworking due to differing home office conditions. As such, this study offers a multi-contextual approach to the investigation of work fatigue and can inform strategies on how to best implement teleworking post-pandemic. This can help to ensure that any future, more permanent changes to teleworking policies include the physical environment and are supportive of employees and organisations.

## Data availability statement

The raw data supporting the conclusions of this article will be made available by the authors, without undue reservation.

## Ethics statement

Ethical review and approval was not required for the study on human participants in accordance with the local legislation and institutional requirements. The participants provided their written informed consent to participate in this study.

## Author contributions

CW, SG, JY, RL, ER, FM, and LW: study conceptualisation and design. CW: data collection, manuscript draft preparation, supervision, and project administration. CW: data curation. KT and CW: formal analysis and visualisation. CW, SG, KT, ER, and TW: editing. All authors contributed to the article and approved the submitted version.

## Funding

Open Access Funding was provided by ZHAW Zurich University of Applied Sciences.

## Conflict of interest

The authors declare that the research was conducted in the absence of any commercial or financial relationships that could be construed as a potential conflict of interest.

## Publisher’s note

All claims expressed in this article are solely those of the authors and do not necessarily represent those of their affiliated organizations, or those of the publisher, the editors and the reviewers. Any product that may be evaluated in this article, or claim that may be made by its manufacturer, is not guaranteed or endorsed by the publisher.

## Footnotes

^1^There is a conceptual and psychometric difference between privacy fit and work-privacy/privacy-work interference; the latter refers to work-family/family-work interference (Meyer et al., 2021).

^2^There is a conceptual and psychometric difference between parental burnout and job burnout (*cf.* Mikolajczak et al., 2020).

^3^The age limit of 15 years was deemed appropriate as the age of legal responsibility and liability as well as maturity concerning various aspects ranges between 14 and 16 years in the primary countries (10–16 years in Switzerland; 14 years in Germany; 10–16 across the United Kingdom; CRIN, 2019). Hence, it was assumed that children from the age of 15 years onwards would require less supervision than younger children.
